# Prognostic Factors of Radiofrequency Ablation plus Systemic Chemotherapy for Unresectable Colorectal Cancer with Liver Metastasis

**DOI:** 10.1155/2020/8836922

**Published:** 2020-12-17

**Authors:** Ky Thai Doan, Long Nguyễn Việt, Thinh Nguyen Tien, Binh Nguyen Canh, Hoai Ngo Thi, Ngoc Nguyen Thanh, Bieu Bui Quang, Quang Le Van, Hyun Woong Lee, Bang Mai Hong

**Affiliations:** ^1^Department of Gastroenterology and Hepatology, 108 Military Central Hospital, Hanoi, Vietnam; ^2^Department of Medical Oncology, 108 Military Central Hospital, Hanoi, Vietnam; ^3^Department of Radiation Oncology and Radiosurgery, 108 Military Central Hospital, Vietnam; ^4^Department of Oncology, Hanoi Medical University Hospital, Vietnam; ^5^Department of Internal Medicine, Yonsei University College of Medicine, Gangnam Severance Hospital, Seoul, Republic of Korea

## Abstract

**Introduction:**

Survival outcomes in patients with unresectable colorectal cancer (CRC) liver metastasis treated by radiofrequency ablation (RFA) combined with systemic chemotherapy and correlation with potential prognostic factors were investigated. *Material and Methods*. A retrospective cohort study was conducted on 61 CRC patients with unresectable liver metastasis who underwent liver tumor-directed percutaneous RFA combined with conventional systemic chemotherapy between October 2013 and September 2018. Survival analyses were conducted using the Kaplan-Meier method, and the log-rank test was used to characterize differences in the median survival time and the 1-year, 3-year, and 5-year overall survival rates of subgroups to identify prognostic factors.

**Results:**

Median overall survival and progression-free survival of all patients were 32 and 14 months, respectively. The cumulative survival rates at 1-, 3-, and 5-years were 93.2%; 44.5%, and 38.2%, respectively. Univariate analysis revealed that pre-RFA serum CEA levels, Eastern Cooperative Oncology Group (ECOG) status, number of liver lesions, the size of the largest lesion, and the total lesion size were prognostic factors. However, multivariate analysis demonstrated that only the number of liver lesions and the size of the largest lesion were independent prognostic factors for survival.

**Conclusion:**

RFA plus systemic chemotherapy provides an encouraging survival outcome for patients with unresectable CRC liver metastasis. Multivariate analysis demonstrated that the number and size of liver metastatic lesions are independent prognostic factors for survival.

## 1. Introduction

Colorectal cancer (CRC) is the third most common cancer by incidence and the second leading cause of cancer-related deaths in both sexes [1]. The incidence of the disease in Vietnam has increased recently [2]. Synchronous liver metastasis reportedly occurs in approximately 20–25% of CRC patients at the time of initial diagnosis. Moreover, 20–30% of patients progress to experience liver metastasis within the first three years after CRC diagnosis. Liver metastasis is considered the main cause of death in CRC patients [3, 4].

Systemic chemotherapy is the standard of care for CRC patients with liver metastasis. However, liver metastatic lesions typically respond poorly to chemotherapy alone; thus, survival outcomes are suboptimal despite the recent introduction of novel chemotherapeutic agents and targeted therapies. Although liver resection is the most curative treatment for liver metastasis, only about 20–25% of CRC patients with liver metastasis are eligible for resection. Radiofrequency ablation (RFA) is an alternative nonsurgical treatment for CRC liver metastasis when the liver metastatic lesions are not eligible for resection or because of comorbidities. However, data on the efficacy of RFA for the treatment of CRC liver metastasis is still limited. Several clinical studies demonstrated that RFA is an effective and safe treatment for liver metastasis in CRC; however, local and distant recurrent rates remain high. The combination of RFA and systemic chemotherapy has also been evaluated in some clinical studies and resulted in promising outcomes for CRC liver metastasis as demonstrated by progression-free survival (PFS) and overall survival (OS). However, the treatment outcome of treated patients is still heterogenous; we are still in the lack of given factors that can be used for prognosing the responses of patients [5–16]. Therefore, the aim of our study was to evaluate survival outcomes and some potential prognostic factors in patients with CRC liver metastasis treated by using RFA combined with systemic chemotherapy.

## 2. Materials and Methods

### 2.1. Patient Selection and Study Design

This retrospective cohort study included 61 consecutive CRC patients with liver metastasis who underwent percutaneous RFA combined with systemic chemotherapy at 108 Military Central Hospital, Hanoi, Vietnam, between October 2013 and December 2018. The study was performed in accordance with the Declaration of Helsinki (1975) and was approved by the Institutional Review Board of 108 Military Central Hospital (O-62720149). As this study was retrospective in nature, written consent from the patients was not required.

The inclusion criteria for this study were as follows: (1) colorectal adenocarcinoma confirmed by histopathology after colorectal resection, (2) liver metastasis confirmed by typical imaging diagnosis (intrahepatic hypo/isoattenuating local lesions with peripheral enhancement in arterial phase and washout in portal venous phase on contrast MDCT, along with high FDG uptake on PET/CT: SUV > 3) [10–12] or liver biopsy, (3) 1–5 liver lesions, (4) maximum size of the largest liver lesion < 5 cm, (5) no clear evidence of extrahepatic spreads confirmed by chest/abdominal CT scans and whole-body PET/CT scan, (6) aged 18–80, and (7) Eastern Cooperative Oncology Group (ECOG) 0–1 without any severe comorbidities, i.e., heart failure, renal failure, or respiratory failure.

Patients were excluded if they had previous liver interventions or surgical liver resection, portal vein thrombosis, presence of active gastrointestinal (GI) bleeding, and severe coagulation abnormalities (prothrombin < 60%, platelet count < 50 G/l). Pregnant or feeding women were also excluded.

All patients were discussed in a multidisciplinary tumor board that included medical oncologists, liver surgeons, radiologists, and hepatologists who performed RFA. The number and the location of the liver lesions, the presence or absence of extrahepatic metastasis, and comorbidities were taken into consideration when determining treatment approaches. Patients deemed to have resectable liver metastasis with severe comorbidity were considered unsuitable for surgery.

### 2.2. RFA Procedure and Chemotherapy Regimens

The Cool-tip RF Ablation System E Series (USA) with multiple probe approaches was used for the RFA technique and performed by two hepatologists who had more than 5 years of experience in percutaneous local ablation for primary and secondary liver cancer. The intervention was conducted percutaneously with ultrasound guidance, and all patients were given local and intravenous anesthesia. Each lesion was calculated to be ablated with at least a 5 mm ablative margin. When lesions were located in difficult locations (i.e., near gastrointestinal tract), an artificial-ascites RFA procedure was used to avoid complications. Tumor necrosis was evaluated by contrast-enhanced CT scan 2 weeks after intervention. A second additional RFA was conducted in patients with incomplete tumor ablation. Systemic chemotherapy was given to patients 2–4 weeks after RFA intervention when all RFA-related adverse events had resolved, and complete ablation of liver lesions was confirmed. FOLFOX-4 or FOLFIRI regimens were chosen for each patient depending on which systemic treatment the patient received for the primary cancer (FOLFOX-4 for first line and FOLFIRI for second line chemotherapy).

Detail of chemo regimens [13]:

- FOLFOX-4: oxaliplatin 85 mg/m2 intravenous (IV) infusion over 2 hours day 1; folinic acid 200 mg/m^2^ IV infusion over 2 hours day 1 + 2; fluorouracil 400 mg/m2 IV bolus day 1 + 2 and fluorouracil 60 0 mg/m^2^ IV infusion over 22 hours day 1 + 2. Total of 12 cycles in every 2 weeks
- FOLFIRI: irinotecan 180 mg/m2 IV infusion over 90 minutes concurrently with folinic acid 400 mg/m^2^ IV infusion over 120 minutes, followed by fluorouracil 400 mg/m^2^ IV bolus, then fluorouracil 2400 mg/m^2^ IV infusion continuously over 46 hours. Total of 12 cycles in every 2 weeks

All patients were treated with pre-and postchemotherapy antiemetics, including 5-HT3 blockers and dexamethasone.

Patient tolerance to chemotherapy was evaluated at the time of the next cycle to adjust the dose of chemo drugs. Follow-up images and serum CEA levels were obtained every 3 months after the first chemotherapy cycle to assess recurrence or progression. Patients with evidence of any recurrence or progression would receive further treatments or just palliative care, after being reevaluated by a multidisciplinary tumor board.

### 2.3. Primary Endpoint

The primary endpoint of this study was OS, defined as the date of RFA for liver metastases to the date of death. Patients lost to follow-up were censored. The second endpoint was progression-free survival (PFS), defined as the date of RFA to the date of disease progression or death.

### 2.4. Statistical Analysis

Baseline characteristics were described as numbers (%) and mean with standard deviation. Clinical variables included age, gender, ECOG, primary cancer, type of metastasis, number of liver metastatic lesions, size of the largest liver lesion, sum of lesion diameters, the type of chemotherapy, and CEA levels. Chi-square or Fisher's exact tests were used to compare count data. The Mann–Whitney *U* test was used to compare two continuous variables with skewed distribution. Kaplan–Meier curves were constructed to examine OS and PFS. We assessed potential predictors of survival using univariate and multivariate Cox proportional hazard regression analyses. The analysis was performed using IBM SPSS Statistics for Windows, version 20.0 (SPSS GmbH, Munich, Germany). Significant differences were defined as *p* < 0.05.

## 3. Results

### 3.1. Baseline Clinical Characteristics

Sixty-one consecutive CRC patients with liver metastasis were recruited for this study. Baseline characteristics of study participants are listed in Table 1. The median follow-up duration was 24 months, the mean age was 56 years (range: 27–71), and there were more males than females (*n* = 49, 80.3% vs. 12, 19.7%). All patients were ECOG 0 or 1. Thirty-seven patients (60.7%) were diagnosed with rectal cancer, and there were a total of 166 liver metastatic lesions treated with 78 RFA sessions. A total of 17 patients were treated with an additional RFA due to incomplete ablation after the first treatment, and they all had complete liver lesion ablation after the second treatment. Artificial ascites-assisted RFA was used in 40/61 patients (65.6%) who had liver metastatic lesions located at the dome of the liver or near the GI tract.

All patients underwent twelve cycles of chemotherapy (40 patients received FOLFOX-4 regimen and 21 others were treated with FOLFIRI regimen). During follow-up, 16/61 (35.6%) of the patients had intrahepatic recurrence, including 7 patients with local tumor progression and 9 patients with new lesions; 18/61 (40.0%) had extrahepatic progression.

### 3.2. Treatment Outcomes

At the time of analysis, 24 patients had died leaving 37 alive. In all cases, the cause of death was disease progression. Kaplan Meier estimations for patient survival demonstrated that (1) median (mean) overall survival time of all patients was 32 (37) months (95% confidence interval (CI): 25–39) and (2) progression-free survival was 14 months (95% CI: 11–17). The cumulative survival rates at 1-, 3-, and 5-year follow-up were 93.2%; 44.5%, and 38.2%, respectively. The median survival time and survival rates of subgroups are presented in Table 2.

**Figure 1:**
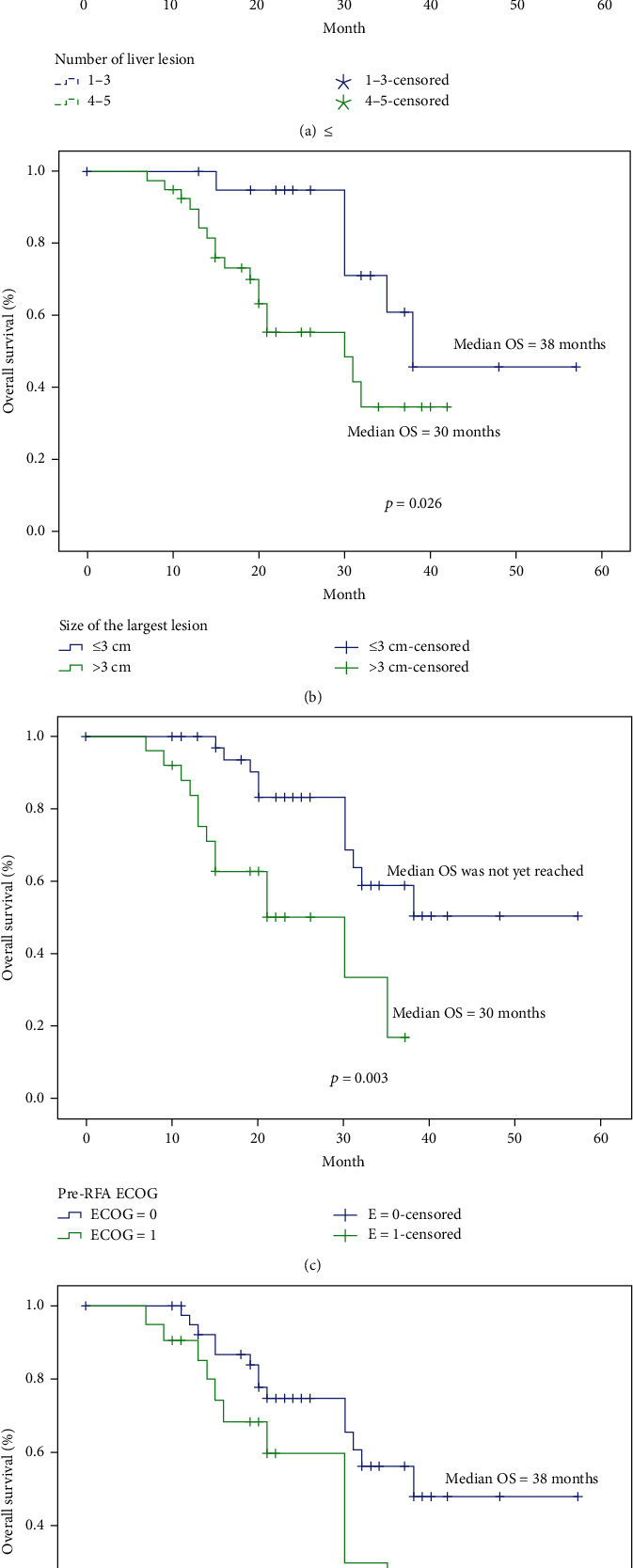
Survival rate subgroup analysis based on number of lesions in the liver (a), size of the largest liver lesion (b), ECOG status (c), pre-RFA serum CEA status (d).

Univariate analysis revealed that the number of liver lesions, the size of the largest lesion, the sum of lesion diameter, and pre-RFA ECOG status were prognostic factors for overall survival (Figure 1). Patients with 1–3 liver lesions had an improved median OS, compared with patients who had 4–5 liver tumors (41 months vs. 30 months, *p* = 0.008). Patients in whom the size of the largest liver lesion was <3 cm also had better median OS, compared with those in whom the largest liver lesion was >3 cm (38 months vs. 30 months, *p* = 0.026). Significant differences in median OS was also demonstrated based on ECOG status and levels of serum CEA (*p* < 0.05). Notably, age, sex, primary cancer, type of metastasis, and chemo regimen criteria did not correlate significantly with patient survival after treatment. A subsequent multivariate analysis revealed that the number of liver lesions (hazard ratio [HR] for 1–3 vs. 4–5, 2.91; confidential interval, 1.1–7.4; *p* = 0.02) and the size of the largest liver lesion (hazard ratio [HR] for ≤3 cm vs. 3–5 cm, 4.72; confidential interval: 1.54–14.4; *p* = 0.01) were independent predictors of OS (Table 3).

### 3.3. Complications

Post-RFA adverse events included right upper quadrant pain (100%), fever (8/61–13.1%), vomiting (4/61–6.6%), and elevated liver enzymes (100%); however, all were mild and resolved within 2 weeks. Two patients experienced pleural effusion after RFA, which resolved after a few days; no other complications or deaths related to intervention were reported.

## 4. Discussion

Although this was not a randomized study, the survival outcomes of the CRC patients with liver metastasis reported here were encouraging considering historical controls treated with systemic chemotherapy alone. It should be noted that even with the recent approval of new chemo regimens, the survival of patients with metastatic CRC has improved modestly to roughly 18–24 months. Several previous studies of patients with CRC liver metastasis have reported improved survival (37–45 months) when systemic chemotherapy was combined with local interventions, but outcomes were different in the literature review. Shuangyan et al. retrospectively evaluated the long-term survival of 109 CRC patients with liver metastasis treated by RFA plus systemic chemotherapy in China and demonstrated survival outcomes similar to those reported here: (1) median survival of 39 months and (2) 1-year, 3-year, and 5-year survival rates of 92.3%; 50.7%, and 41.6%, respectively [14]. Rues et al. reported the results of a randomized phase II study (EORTC 40004) characterizing RFA combined with systemic chemotherapy for unresectable colorectal liver metastasis. Patients were randomized into one of two treatment arms: (I) systemic chemotherapy alone (FOLFOX/FOLFIRI plus bevacizumab) or (II) systemic chemotherapy plus RFA for liver lesions. The median overall survival of patients in arm I was 40.5 months, compared with 45.3 months for arm II combined treatment. The difference was not significant (*p* = 0.22). However, RFA plus systemic treatment resulted in significantly longer PFS (16.8 months vs. 9.9 months, *p* = 0.025) [15]. Survival of patients in the EORTC 40004 study, which was better than survival in the study being described here, may be explained by the addition of bevacizumab to systemic treatment and longer follow-up. Bevacizumab is an expensive therapy and not fully covered by government reimbursement; therefore, it is currently not routinely used in clinical practices [16, 17]. Better survival outcomes were also seen in a study of Solbiati et al. with a median overall survival of 53 months, but the patient population included only small, favorably located liver metastasis (i.e., maximum diameter of any metastasis < 4 cm, each >1 cm away from hepatic hilum or GI tract) with no extrahepatic disease, and a proportion of these patients was eligible for resection [18]. Meanwhile, another prospective study by Berber et al. in the United States, involving 135 patients with CRC liver metastasis treated by laparoscopic RFA combined with systemic chemotherapy, demonstrated a median overall survival of only 28.9 months, shorter than that in our study. Berber's study recruited older patients (mean age 62 years old), more patients with previous chemotherapy exposure (108/135), and patients with extrahepatic disease, which may explain the outcome [19]. Patient survival in the current study was also higher than that in a study conducted by Gillams et al. (median survival of 27 months; 1-, 3-, and 4-year survival of 90%, 34%, and 22%, respectively). However, Gillams and colleagues included patients across a broad spectrum of disease severity: (1) did not exclude those with extrahepatic metastasis, (2) the number of liver lesions was 1–16, and (3) the diameters of lesions were 1–8 cm [20].

The effectiveness of RFA plus systemic chemotherapy in improving survival varies; thus, an analysis of potential prognostic factors of posttreatment survival is warranted. A multivariate analysis demonstrated that the number of liver lesions and size of the largest lesion were both independent predictors of patient survival. In the study by Berber et al., the size of the largest liver lesion and serum CEA levels was significantly related to the overall survival of patients. Patients with a serum CEA less than 200 ng/ml had improved survival compared with those with a CEA more than 200 ng/ml (34 vs. 16 months; *p* = 0.01). The median survival varied depending on the diameter of the largest liver lesion: (1) 38 months (<3 cm), (2) 34 months (3–5 cm), and (3) 21 months (>5 cm) (*p* = 0.03), and the presence of extrahepatic disease did not affect survival [19]. Shady et al. evaluated factors affecting outcomes of percutaneous RFA of CRC liver metastasis in a retrospective single-center study with 10 years of follow-up; using univariate analysis, the authors demonstrated that the size of the largest liver lesion (greater or less than 3 cm), serum CEA level (greater or lower 30 ng/ml), and presence or absence of extrahepatic diseases were independent predictors for overall survival. However, multivariate analysis revealed that only lesion size greater than 3 cm and more than one site of extrahepatic diseases was independent predictors of shorter overall survival [21]. In the analysis reported here, no extrahepatic spreads or lymph node metastases were present, an observation confirmed by chest/abdominal CT scan or whole-body PET/CT.

Seifert and Morris investigated potential prognostic factors in 116 patients with CRC liver metastasis undergoing cryotherapy and reported that low serum CEA levels (<5 ng/ml), small (≤3 cm) diameter of liver metastasis, absence of untreated extrahepatic disease at laparotomy, absence of nodal involvement at primary resection, complete cryotherapy, synchronous development of liver metastasis, and good or moderate differentiation of primary tumors were independently associated with a favorable outcome [22]. The liver-tumor burden impacts survival in this study, an observation that is corroborated by other studies. This can be explained by the risk of microscopically incomplete ablation in patients with higher liver tumor burden. We should note that the liver tumor burden is also a very important risk factor for survival in patients with CRC liver metastasis after resection [23, 24].

This study has some limitations. The small patient population and the retrospective nature of the study are potential drawbacks in that it might be insufficient to guide decisive conclusions on the other potential prognostic factors tested. However, the results of our study suggest that at least two parameters (i.e., total lesion number and size of the largest lesion) should be taken into account when considering RFA plus systemic chemotherapy for CRC patients with liver metastasis. The integration of these metastatic tumor characteristics in predicting the long-term survival of patients with CRC liver metastasis should also be addressed.

In conclusion, RFA plus systemic chemotherapy demonstrates an encouraging survival outcome for patients with unresectable CRC liver metastasis. The number of liver metastatic lesions ≤ 3 and size of the largest lesion ≤ 3 cm were the only independent prognostic factors of better posttreatment survival identified.

## Figures and Tables

**Table 1:** Baseline characteristics of study participants (*n* = 61).

Variables	*n* = 61 (%)
Male	49 (80.3)
Mean age (years)	55.57 ± 9.40 (27-75)
ECOG	
0	25 (41)
1	36 (59)
Primary cancer	
Colon	24 (39.3)
Rectum	37 (60.7)
Type of metastasis	
Synchronous	24 (39.3)
Metachronous	37 (60.7)
Adjuvant chemotherapy for primary cancer	
Yes	29 (47.5)
No	32 (52.5)
Number of liver metastatic tumors	166^∗^ (2.72 ± 1.34/patient)
1–3	41 (67.2)
4–5	20 (32.8)
Size of the largest liver lesion	
≤3 cm	21 (34.4)
> 3 cm	40 (65.6)
Sum of lesion diameters	^†^7.20 ± 3.95 cm
<5 cm	26 (42.6)
5–10 cm	21 (34.4)
>10 cm	14 (23.0)
Serum CEA	
≤30 ng/mL	40 (65.6)
>30 ng/mL	21 (34.4)

ECOG: Eastern Cooperative Oncology Group. ^∗^Total number of liver metastatic lesions. ^†^Mean sum of tumor diameters in a patient.

**Table 2:** Estimated survival times by potential prognostic factor.

Variables		Median survival, months (95% CI)	*p* value
Sex	Male (*n* = 49)	35 (27–42)	0.155
	Female (*n* = 12)	32 (27–39)	
Age	<40 (*n* = 5)	40 (21–58)	0.66
	40–60 (*n* = 35)	32 (25–38)	
	>60 (*n* = 13)	37 (31–42)	
Number of liver lesions	1–3 (*n* = 41)	41 (35–48)	0.008
	4–5 (*n* = 20)	24 (19–29)	
Size of the largest liver lesion	≤3 cm (*n* = 21)	38 (35–51)	0.026
	>3 cm (*n* = 40)	30 (15–44)	
Sum of the diameter of liver lesions	<5 cm (*n* = 23)	43 (35–51)	0.011
	5–10 cm (*n* = 24)	30 (25–36)	
	>10 cm (*n* = 14)	22 (16–27)	
Primary cancer	Colon (*n* = 37)	40 (33–47)	0.263
	Rectum (*n* = 24)	28 (23–33)	
Type of metastasis	Synchronous (*n* = 24)	32 (28–35)	0.69
	Metachronous (*n* = 37)	35 (29–50)	
Chemo regimen	FOLFOX–4 (*n* = 40)	32 (23–46)	0.346
	FOLFIRI (*n* = 21)	28 (23–33)	
ECOG performance status	0 (*n* = 36)	42 (36–49)	0.003
	I (*n* = 25)	24 (19–29)	
Serum CEA	≤30 ng/mL (*n* = 40)	40 (33–46)	0.035
	>30 ng/mL (*n* = 21)	25 (20–30)	

ECOG: Eastern Cooperative Oncology Group; CEA: carcinoembryonic antigen.

**Table 3:** Multivariate analysis of independent prognostic factors for overall survival.

Variables		Multivariate analysis	
		HR (95% CI)	*p* value
Age	<40	1.63 (0.7–3.6)	0.24
	40–60		
	>60		
Primary cancer	Rectum	0.54 (0.2–1.3)	0.18
	Colon		
Previous adjuvant chemotherapy	No		
	Yes	2.08 (0.5–7.7)	0.26
Number of liver lesions	1–3	2.91 (1.1–7.4)	0.02
	4–5		
Size of the largest liver lesion	≤3 cm	4.72 (1.54–14.4)	0.01
	3–5 cm		
Chemo regimen	FOLFOX4		
	FOLFIRI	1.15 (0.36–3.66)	0.88
Serum CEA > 30 ng/mL	No	1.73 (0.6–4.5)	0.25
	Yes		

CEA: carcinoembryonic antigen.

## Data Availability

No data used to support the findings of the study.

## Abbreviations

CRC: Colorectal cancer
RFA: Radiofrequency ablation
PFS: Progression-free survival
OS: Overall survival
GI: Gastrointestinal
ECOG: Eastern Cooperative Oncology Group.
